# A multi‐contrast MRI approach to thalamus segmentation

**DOI:** 10.1002/hbm.24933

**Published:** 2020-01-20

**Authors:** Veronica Corona, Jan Lellmann, Peter Nestor, Carola‐Bibiane Schönlieb, Julio Acosta‐Cabronero

**Affiliations:** ^1^ Department of Applied Mathematics and Theoretical Physics University of Cambridge Cambridge UK; ^2^ Institute of Mathematics and Image Computing University of Lübeck Lübeck Germany; ^3^ Queensland Brain Institute University of Queensland Brisbane Queensland Australia; ^4^ Mater Hospital South Brisbane Queensland Australia; ^5^ Wellcome Centre for Human Neuroimaging, UCL Institute of Neurology University College London London UK; ^6^ German Center for Neurodegenerative Diseases (DZNE) Magdeburg Germany

**Keywords:** magnetic resonance imaging, multi‐contrast segmentation, thalamic nuclei, thalamus segmentation

## Abstract

Thalamic alterations occur in many neurological disorders including Alzheimer's disease, Parkinson's disease and multiple sclerosis. Routine interventions to improve symptom severity in movement disorders, for example, often consist of surgery or deep brain stimulation to diencephalic nuclei. Therefore, accurate delineation of grey matter thalamic subregions is of the upmost clinical importance. MRI is highly appropriate for structural segmentation as it provides different views of the anatomy from a single scanning session. Though with several contrasts potentially available, it is also of increasing importance to develop new image segmentation techniques that can operate multi‐spectrally. We hereby propose a new segmentation method for use with multi‐modality data, which we evaluated for automated segmentation of major thalamic subnuclear groups using *T*_1_‐weighted, $T_{2}^{*}$‐weighted and quantitative susceptibility mapping (QSM) information. The proposed method consists of four steps: Highly iterative image co‐registration, manual segmentation on the average training‐data template, supervised learning for pattern recognition, and a final convex optimisation step imposing further spatial constraints to refine the solution. This led to solutions in greater agreement with manual segmentation than the standard Morel atlas based approach. Furthermore, we show that the multi‐contrast approach boosts segmentation performances. We then investigated whether prior knowledge using the training‐template contours could further improve convex segmentation accuracy and robustness, which led to highly precise multi‐contrast segmentations in single subjects. This approach can be extended to most 3D imaging data types and any region of interest discernible in single scans or multi‐subject templates.

## 1 INTRODUCTION

The thalamus is composed of a complex set of sub‐nuclei. It is considered the central relay station for sensory and motor information as nearly all sensory and motor signals are sent to the thalamus prior to reaching the cortex. It is also thought to have an integrative role as thalamic structures receive, process, sort and send information between specific subcortical and cortical areas, and might be involved in regulation of sleep and wakefulness, memory, emotion, consciousness, awareness, attention, ocular motility, learning and motor control processes (Jürgen, 2011; Sherman & Guillery, 2002; Steriade & Llinás, 1988).

The thalamus is composed of several major substructures. The internal medullary lamina is a thin sheet of white matter that runs longitudinally through the thalamus separating it into medial and lateral regions. In the anterior part, the internal medullary lamina forks to isolate the anterior thalamic nucleus; thus, thalamic nuclei can be broken down into four regions based on their position relative to the lamina, that is, anterior, medial, lateral and posterior subnuclear groups (Chien, Cheng, & Lenz, 2016; Michael Conn, 2016).

Lesions to thalamic nuclei and their connections to the cortex can result in a wide range of neurological deficits. Thalamic alterations have been identified in several neurodegenerative diseases including Alzheimer's disease, Parkinson's disease, Huntington's disease and multiple sclerosis, the majority of which present evidence of atrophy in one or more substructures of the thalamus (Amano, 2004; Kassubek, Juengling, Ecker, & Landwehrmeyer, 2005; Power & Looi, 2015; Steriade & Llinás, 1988). Neurological patients also often undergo brain surgery and deep brain stimulation targeting thalamic subnuclei, thus accurate and reliable localisation of such structures are key for both research and delivering effective clinical treatments (Ondo, Almaguer, Jankovic, & Simpson, 2001; Steriade & Llinás, 1988).

New developments in imaging techniques, including 3–7 Tesla MRI, provide greater contrast and higher spatial specificity to study the thalamus. Therefore, new strategies need to be investigated for clinical and research applications, which could potentially lead to suitable tools for predicting cognitive impairment and for monitoring disease progression in neurological patients (Gringel et al., 2009).

To date, several methods have been proposed to delineate subthalamic regions with MRI, a few of which used diffusion MRI. For example, Behrens et al. (2003) described a diffusion tensor imaging (DTI) based segmentation procedure based on coarse tractography patterns from the thalamus to the cortex, and Wiegell, Tuch, Larsson, and Wedeen (2003) developed a k‐means clustering algorithm to detect groups of coherent DTI‐based fibre orientation. Lambert et al. (2012) identified three main regions on diffusion weighted imaging (DWI) using a clustering algorithm based on a probabilistic index of connectivity. The use of the mean‐shift algorithm (Duan, Li, & Xi, 2007) has also been proposed, whereby regional clusters and shapes are inferred from the local modes of a density estimator computed with a multivariate kernel (Duan et al., 2007). Furthermore, Jonasson et al. (2007) proposed a level‐set method whereby a region‐based force (defined using a diffusion similarity index between the most representive tensor of each level set and its neighbours) drives a set of coupled level‐set functions each representing a segmented region. High angular resolution diffusion images (HARDI) have also been investigated for segmenting the thalamus. Grassi et al. (2008) proposed a k‐means clustering approach whereby a specific number of initialised centroids are updated based on a weighted sum of the Euclidean distance of voxels to centroids and Frobenius distance of their orientation distribution function. Notably, however, all diffusion MRI based methods are hampered by low spatial resolution. In an attempt to overcome this limitation, Deoni, Rutt, Parrent, and Peters (2007) explored with some success the use of high‐resolution quantitative MRI, namely, *T*_1_ and *T*_2_ mapping, with a modified k‐means clustering approach that combined *T*_1_/*T*_2_ information and center‐of‐mass distances to Morel atlas segmentations (Morel, Magnin, Jeanmonod, et al., 1997). Further using anatomical images, Magon et al. (2014) developed a method to segment thalamic subnuclei using a voxel‐wise majority vote after co‐registration to multiple atlases.

Past efforts also focused on the MRI acquisition. Bender, Mänz, Korn, Nägele, and Klose (2011), for example, proposed an inversion time optimisation strategy to enhance the *T*_1_‐weighted contrast between grey and white matter using the 3D magnetization‐prepared rapid acquisition of gradient echo (MPRAGE) sequence. Tourdias, Saranathan, Levesque, Su, and Rutt (2014) subsequently optimised MPRAGE for 7 T MRI and proposed imaging at the white matter null regime both for enhancing the contrast between the thalamus and surrounding tissues and for depicting several subnuclear groups.

Thalamus segmentation with quantitative susceptibility mapping (QSM)—a relatively new quantitative MRI contrast—has also gained increasing interest in recent times. Deistung et al. (2013) illustrated that high‐resolution QSM is a superior contrast to depict thalamic substructures than $T_{2}^{*}$‐weighting, the local field or $R_{2}^{*}$ maps. Therefore, considering QSM's ability to provide quantifiable information about iron content (Hametner et al., 2018), that iron accumulation has been associated with several neurological disorders (Ward, Zucca, Duyn, Crichton, & Zecca, 2014) and that thalamic lesions are not uncommon in such disorders (Kassubek et al., 2005; Steriade & Llinás, 1988), it is highly plausible that enabling reliable segmentation of thalamic substructures with multiple MRI contrasts including QSM could have a major impact on the study of ageing and disease.

Traditionally, however, the anatomy of the thalamus has been inferred from post‐mortem tissue examinations. The most widely used histological atlas was developed by Morel et al. (Morel et al., 1997) using an iterative approach for reconstructing the mean model from six series of maps derived from different stacks of histologically processed brains. The model, thus, is an average template incorporating topological and geometric features from only a few individuals. More recently, Ilinsky et al. (2018) derived a model for the thalamus, with focus on parcellation of connectivity distinct motor‐related nuclei, which identified major subcortical afferent zones. A probabilistic atlas of thalamic nuclei has also been proposed by Iglesias et al. (2018). The atlas was derived combining ex vivo MRI and histological data from six autopsy samples, and it can be applied to in vivo MR images for segmentation by solving a Bayesian inference problem.

Morel's and other similar proprietary atlases are widely used for guiding MRI‐based segmentations in neurosurgical planning, although notably, the direct superposition onto brain scans is often not fit for precision measurements, a situation often aggravated by age‐related differences (Steriade & Llinás, 1988). Therefore, the development of image‐guided segmentation approaches is highly relevant in this context.

### 1.1 Our contribution

**Figure 1:**
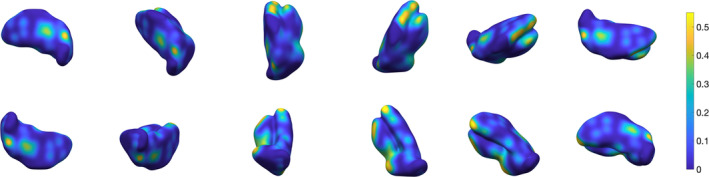
Three‐dimensional rendering of the thalamus segmentation using our multi‐contrast approach. The surface is colour‐coded by average absolute distance with respect to ground truth's outer boundary

This work proposes a new multi‐contrast segmentation algorithm, and its optimisation, to exploit the full potential of *T*_1_‐, $T_{2}^{*}$‐weighted and QSM contrast differences in thalamic subregions. We show that using multi‐contrast information maximises segmentation performance, by exploiting structures that become visible and enhanced in specific MR imaging contrasts. In the proposed method, regions of interest defined in template space are learnt and then approximated in single subjects with spatial constraints that ensure robustness. Our multi‐contrast segmentation framework can be extended to any data types and regions of interest. In Figure 1, we show a preview of the output of our approach. In this 3D view, the surface, which is colour‐coded by average absolute distance error with respect to the ground truth's outer boundary, illustrates that local error modes are typically of the order of <10%. In the following sections, we provide a detailed description of the proposed method, and optimization as well as validation results.

## 2 METHODS

The proposed semi‐automated method consists of four steps: Spatial normalisation, manual (reference) segmentation, pattern recognition and a final refinement step using a convex formulation.

Details on study subjects, MRI acquisition and pre‐processing are given below. For now, we will assume all subject data has been spatially co‐registered to a common reference space, from which multi‐subject templates (one for each contrast) have been computed. We will also assume hereafter (specific details given below) that regions of interest have been manually traced (at least once) with the aid of such templates. We then consider the following multi‐class labelling problem.

### 2.1 Classification

For each voxel in the image volume domain Ω ⊂ ℝ^3^, Ω = {1, …, *n*_1_} × {1, …, *n*_2_} × {1, …, *n*_3_}, we assign one of *ℓ* class labels, with each class referring to a segmented region. Let *X* = {*x*_*i*_, *i* = 1, …, *n*}, where *n* = *n*_1_ *n*_2_ *n*_3_, be the vectorised volume in template space. For each *x*_*i*_, we have *c* image intensities or MRI parameter values, *f*_1_(*x*_*i*_), …, *f*_*c*_(*x*_*i*_), one from each imaging contrast available; in this study, *T*_1_‐, $T_{2}^{*}$‐weighted signals and QSM. We then identify a set of possible class labels, {0, 1, …, ℓ − 1}; in this particular context, we set 0 to be the background region, 1 the lateral thalamic subnuclear group, 2 the medial group and 3 the posterior group. The manual segmentation in template space is required to define the label set for the volume *X* as *Y* = {*y*_*i*_, *i* = 1, …, *n*}, where *y*_*i*_ ∈{0, 1, 2, 3}.

### 2.2 Feature space

In the reference coordinate system, we then build the feature space: Ψ = {*ψ*_*ij*_, *i* = 1, …, *n*; *j* = 1, …, *m*}, assigning to each voxel *x*_*i*_, *i* = 1, …, *n*, in the image volume, an *m*‐dimensional feature vector. Features describe objects, in our case voxel information reflecting thalamic tissue properties. In this work, we set out to develop a multi‐spectral approach to exploit features from several contrast types, whereby the key features are intensity/MRI parameter values: *f*_*k*_(*x*_*i*_), *k* = 1, 2, 3 from *T*_1_, $T_{2}^{*}$‐weighted MRI and QSM, which return different contrast characteristics for tissues with different local concentrations of water, iron, myelin and so forth. For each contrast, we also selected additional features which are the result of an empirical study of the feature space. These are mean, *μ* and standard deviation, *σ*, across the 26‐neighbourhood and intensity/MRI parameter values for the six closest 3D neighbours in each contrast, leading to a feature space of *m* = 27 dimensions. All features were then scaled by their normalised variance (i.e. with mean shifted to the origin and total variance for all features scaled to 1). It should be noted that this feature space was selected for the present application through an investigation of classification accuracy versus feature space dimensionality on a data subset. This might differ for other data types and/or target regions.

### 2.3 Classifiers

Let us consider the feature space Ψ and the label set *Y* for *n* voxels. Each template voxel is therefore described by the pair (***ψ***_*i*_, *y*_*i*_), where ***ψ***_*i*_ is the *m*‐dimensional feature vector of voxel *x*_*i*_ and *y*_*i*_ is its label. We define the labelled training dataset as $T=\left\{\left(\psi_{1},y_{1}\right),\ldots ,\left(\psi_{n},y_{n}\right)\right\}$. We set out to solve a classification problem based on supervised learning, in which we train a classifier to derive a decision mapping for new observations. Initially, we explored the performance of several classification methods including support vector machine, random forest, Naive‐Bayes, k‐nearest neighbours (*k*‐NN) and Parzen classifiers using a data subset. In this preliminary study, we obtained greater accuracy with two classifiers: *k*‐NN (*k* = 3) and *Parzen* classifiers, which are described in (Theodoridis & Koutroumbas, 2008) and, for the sake of completeness, are summarised in Appendix A. Results for the other classifiers are reported in Appendix B. As pointed out in the context of feature selection, the optimal choice of training classifier may also vary according to data type and/or target region.

### 2.4 Convex segmentation

Classification routines yield a posterior probability distribution $\hat{p}\left(u|f\right)\in {\mathbb{R}}^{n\times \ell }$ for each class, that is the probability for voxel *x* to be assigned class *u*(*x*) = *l* given the measured data *f*(*x*). From this, winner‐takes‐all segmentation can be derived selecting the class with the highest probability value in each voxel. This, however, often results in scattered clusters of misclassified voxels that break the smoothness and continuity of segmented regions. Hereby, therefore, we introduce an additional convex optimisation step to further improve the spatial cohesiveness of tissue segments.

More precisely, we consider a labelling function *u* : Ω → ℝ^ℓ^ that represents the unique assignment of a label to each voxel *x* in the image domain Ω. Because this is a hard combinatorial problem, we use a convex relaxation to facilitate the optimisation, see (Lellmann & Schnörr, 2011) for an overview. The notion of labelling function is relaxed to *u* taking values in the convex set defined by the unit simplex$\Delta_{\ell }≔\left\{u\in {\mathbb{R}}^{n}\to {\mathbb{R}}^{\ell }|u\geq 0,\sum_{i=1}^{\ell }u_{i}=1\right\}$. Then, by choosing *J* convex, we solve the following convex segmentation (CS) problem:

(1) $$\min_{u:\Omega \to \Delta^{\ell }}\underset{\text{data term}}{\underset{︸}{\sum_{x\in \Omega }-\log\hat{p}\left(u\left(x\right)|\left(x\right)\right)}}+\underset{\text{regulariser}}{\underset{︸}{\lambda \mathrm{TV}\left(u\right)}},$$

whereby the data term is the negative logarithm of the posterior probability distribution computed by the classifier, and the regulariser is the total variation (TV) of the labelling function *u* defined as the L^1^‐norm of a discrete finite‐difference approximation of the two‐dimensional gradient (Appendix C).

The TV regulariser on the relaxed *u* is the convex equivalent to the length penalty on the original hardcoded labelling function and, as such, it can be thought of as introducing a penalty for long or irregular interfaces between different classes. The parameter *λ* > 0 balances the data term and the regulariser in the minimisation. We solve (1) using the fast primal‐dual algorithm described in Chambolle, Cremers, and Pock (2012) and Pock, Cremers, Bischof, and Chambolle (2009).

### 2.5 Convex segmentation with additional priors

Individual datasets are overall inferior to group‐wise templates in terms of signal‐ and contrast‐to‐noise ratio. With a view then to ensure segmentation robustness at the single‐subject level, we extended the forward model by the introduction of a priori information on the manual segmentation of the training template. We enabled the weighting of posterior probabilities, $\hat{p}\left(u|f\right)$, according to template‐based constraint as follows:

(2) $$\min_{u:\Omega \to \Delta^{\ell }}\sum_{x\in \Omega }-\log\left(\left(1-w\right)\;\hat{p}\left(u\left(x\right)|f\left(x\right)\right)+w\;m\right)+\lambda \mathrm{TV}\left(u\right)$$

where *w* ∈ [0, 1] is a normalised weight determining the level of prior information constraining the data term, *m*: Ω → {0, 1}^*ℓ*^ is a labelled mask of thalamic subregions, and *λ* > 0 is the regularisation parameter.

### 2.6 Study subjects

Training (Dataset I) and test (Dataset II) datasets consisted of *N* = 43 (age: 59 ± 7, [50–69] years old, 19 female/24 male) and *N* = 116 (age: 54 ± 19, [20–79] years old, 56 female/60 male) healthy subjects, respectively. The latter was an ageing cohort previously investigated with QSM (Acosta‐Cabronero, Betts, Cardenas‐Blanco, Yang, & Nestor, 2016). All elderly subjects (age > 50 years old) were free of neurological or major psychiatric illness and performed normally on cognitive screening: Mini‐mental state examination (MMSE > 27; Folstein, 1975).

### 2.7 MRI scanning protocol

The imaging protocol, QSM reconstruction and spatial normalisation methods (briefly summarised below) are essentially identical to those in a previous ageing study (Acosta‐Cabronero et al., 2016).

All participants were scanned on a Siemens Verio 3 Tesla MRI system with a 32‐channel head coil (Siemens Medical Systems, Erlangen, Germany) under the following imaging protocol:

*T*_1_‐weighed data were acquired using a 3D MPRAGE sequence (Mugler & Brookeman, 1990) with the following scan parameters: Inversion time = 1,100 ms, flip angle (*α*) = 7°, echo time (TE) = 4.37 ms, receiver bandwidth (RB) = 140 Hz per pixel, echo spacing = 11.1 ms, repetition time (TR) = 2,500 ms; 256 × 256 × 192 matrix dimensions (straight‐sagittal orientation), 1 × 1 × 1 mm^3^ voxel size, twofold parallel acceleration and further 7/8 partial Fourier undersampling for phase encoding. The total scan time was 5:08 min.

$T_{2}^{*}$‐/susceptibility‐weighted data were obtained from a fully flow‐compensated, 3D spoiled gradient‐echo sequence. Scan parameters were: *α* = 17^°^, TE = 20 ms, RB = 100 Hz per pixel, TR = 28 ms; matrix, 256 × 224 × 80 with voxel resolution of 1 × 1 × 2 mm^3^; and twofold parallel acceleration for phase encoding. The total scan time was 5:32 min. All susceptibility maps were inspected to exclude subjects with severe calcifications or microbleeds.

### 2.8 MRI data pre‐processing

#### 2.8.1 QSM reconstruction

QSM is a relatively new contrast modulated by the local content of chemical species that have different magnetic susceptibilities than soft tissue and water (Wang & Liu, 2015). Myelin phospholipids and calcifications, for example, are more diamagnetic than water; whereas, iron—the most abundant transition metal in the human brain and the dominant source of QSM contrast—is greatly paramagnetic (Hametner et al., 2018).

Technically, QSM uses complex‐valued information from three‐dimensional (3D) gradient echo MRI sequences. Multi‐channel complex data were combined for optimal phase reconstruction using an adaptive algorithm (Walsh, 2000), preceded by an in‐house algorithm for reference channel selection that automatically identifies the channel with the highest fifth percentile SNR across a brain mask. Combined phase images were then unwrapped with a direct Laplacian approach (Schofield & Zhu, 2003), and the background field induced at tissue–air interfaces was removed using the SHARP filtering method (Schweser, Deistung, Lehr, & Reichenbach, 2011), with a kernel radius of 5 mm. Finally, nonlinear MEDI (Liu et al., 2013), a regularised dipole inversion algorithm, was used to find a unique solution (a) that is model consistent with the input local field and (b) that matches the anatomical structure depicted on magnitude images. Specific details on the susceptibility reconstruction methodology used in this study can be found elsewhere (Acosta‐Cabronero et al., 2016).

#### 2.8.2 Spatial standardisation

Radio‐frequency (RF) bias corrected (Tustison et al., 2010) $T_{2}^{*}$‐weighted magnitude images were affine co‐registered to their corresponding bias‐corrected MPRAGE volume using ANTs (http://stnava.github.io/ANTs/; Avants, Tustison, & Song, 2009). Subsequently, all bias‐corrected anatomical *T*_1_‐weighted MPRAGE images were used to generate a study‐wise space using a previously described ANTs routine (Acosta‐Cabronero et al., 2016; Avants, Epstein, Grossman, & Gee, 2008). Finally, all $T_{2}^{*}$‐weighted images and susceptibility maps were normalised to the same coordinate system through the warp composition of the above transformations and high‐order interpolation, to match the 1 mm isotropic voxel resolution of the MPRAGE volume.

#### 2.8.3 Manual (reference) segmentation

**Figure 2:**
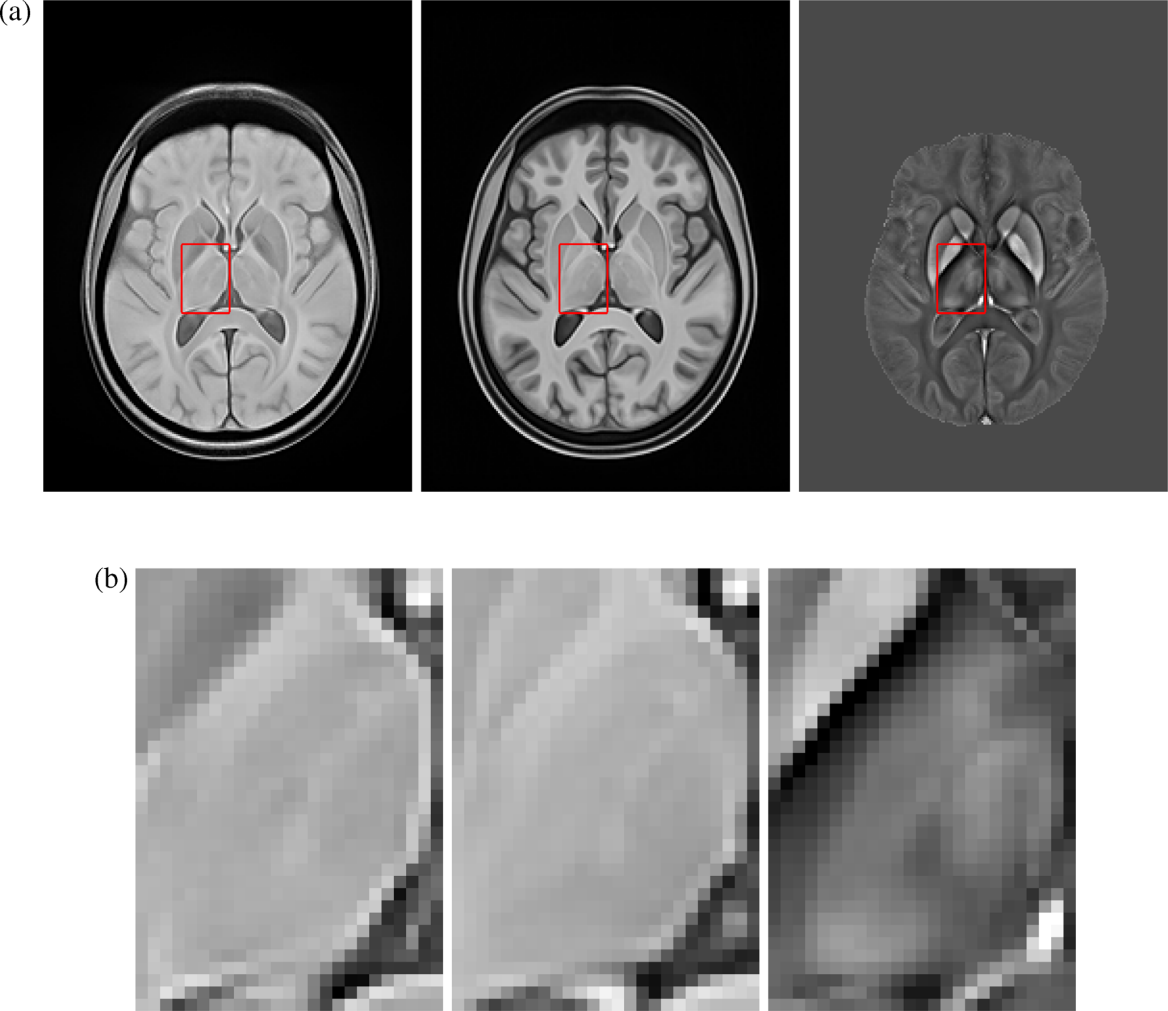
Anatomical detail from group‐average MRI templates. (a) Representative axial cuts (from left to right) of $T_{2}^{*}$‐, *T*_1_‐weighted and QSM templates for the test dataset (Dataset II). (b) Magnified views of the left thalamus for the three templates

Three templates were subsequently obtained from averaging *T*_1_‐, $T_{2}^{*}$‐weighted and QSM data across subjects in the study‐wise space (Figure 2). This was performed separately for training and testing data.

**Figure 3:**
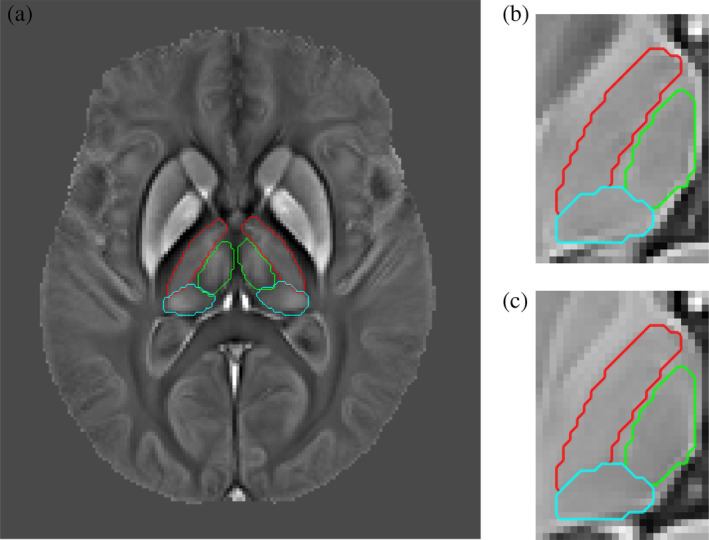
Manual segmentation of three major thalamic subnuclear groups. *Left*: Bilateral contours overlaid onto the Dataset II QSM template. Red contours denote the lateral nuclear group, green contours correspond to the medial group, and cyan contours illustrate the posterior group segmentation. *Right*: Magnified view of the left thalamus showing manual contours overlaid onto $T_{2}^{*}$‐ and *T*_1_‐weighted templates. (a) Manual contour overlays onto the *N* = 116 average QSM template. (b) $T_{2}^{*}$‐weighting (c) *T*_1_‐weighting

Free‐hand segmentations were guided by the three contrasts simultaneously toggling between views in ITK‐SNAP (http://www.itksnap.org). The references throughout were the internal white matter lamina of the thalamus, and other contrast variations consistent with prior knowledge based on the Morel atlas. Three major thalamic subregions, namely, lateral, medial and posterior nuclear groups, were manually traced as illustrated in Figure 3. The manual annotations from the training‐average template were utilised in the training phase of the algorithm as ground truth. In addition, thalamic subregions from the average test template (Dataset II) and for *N* = 16 (ages: 24, 25, 38, 47, 51, 62, 63, 64, 68, 70, 72 and 78) individual test datasets were delineated for algorithm validation (section 2.9), giving intra‐rater Dice scores of 98.23 and 84.89 for template and mean single‐subject segmentation, respectively. Inter‐rater variability was not calculated in this study.

### 2.9 Performance evaluation

Segmentation performance was assessed through visualisation of the confusion matrix (incorporating exact error distributions). For simplicity, however, in this study, we report two representative summary measures: The global classification error rate (i.e. the overall proportion of erroneously classified voxels) and the true positive (TP) rate for non‐background (i.e. subnuclear group) regions. In the first instance, algorithm performance evaluations were carried out for the thalamus nuclear group segmentation from high SNR templates (Dataset II average), including a comparison with standard Morel atlas based segmentation. Subsequently, error measures were also computed for individual (noisier, multi‐contrast) test data.

Morel segmentation was performed using a co‐registration based approach. This used a pre‐optimised routine for unimodal (*T*_1_‐weighted MRI) registration in ANTs (‘antsRegistrationSyN.sh’, http://stnava.github.io/ANTs) incorporating both rigid (multi‐resolution) and non‐linear (multi‐resolution, b‐spline SyN; Avants et al., 2008) transformation steps. This has recently suggested as the state‐of‐the‐art approach in this context (Ewert et al., 2019). Morel atlas labels were obtained from the original source (Krauth et al., 2010) in 0.5‐mm isotropic resolution, MNI152 template (Montreal Neurological Institute, McGill University, Canada) co‐ordinates. The high‐resolution MNI152 template was thus co‐registered to our study‐wise template to map Morel labels onto our study space. N.b. Morel labels were redefined (i.e. merged together where appropriate) to conform the nuclear group labels used in this work.

### 2.10 Methods summary

In Algorithm 1, we summarise the proposed methodology for multi‐contrast MRI segmentation. The first stage of the algorithm trains a classifier for use in stage two. Given then a ‘new’ multi‐contrast MRI dataset to be segmented, all contrast images must be first realigned to a common space, then the ‘new subject’ segmentation pipeline can be applied as follows:

- supervised classification (testing), given the trained classifier, its mapping is applied to independent test data yielding class labels and posterior probabilities.
- multi‐class convex segmentation, where posterior probabilities are used in the data term of the convex optimisation formulation defined in Equation (1).

The supervised learning and convex segmentation steps of the algorithm were implemented in MATLAB R2017b (The Mathworks Inc., Natick, MA) and are available at https://github.com/veronicacorona/multicontrastSegmentation.git.

Algorithm 1 Procedural steps for multi‐contrast segmentation.

Training stage

**Input:** Multi‐contrast training data

**1:** Spatial normalisation

**2:** Contrast‐specific template generation

**3:** Manual (or atlas based) template segmentation

**4:** Supervised classification (training)

**Output:** Trained classifier

New segmentation

**Inputs:** Multi‐contrast test data and a trained classifier

**5:** Spatial normalisation

**6:** Supervised classification (testing)

**7:** Multi‐contrast convex segmentation

**Output:** Regional contours

Remark 1 *All MRI datasets in this study were spatially standardised* via *nonlinear co‐registration to a common coordinate system. This enabled custom training from a single set of regional contours in template space. Future applications of this algorithm could alternatively consider using manual tracings from each individual training dataset. The only requirement is that all contrasts for a given subject must share a common frame of reference*.

## 3 RESULTS

In what follows we present our numerical results obtained independently for Dataset II described in section 2.4 (Sherman & Guillery, 2002).

### 3.1 Qualitative assessment

**Figure 4:**
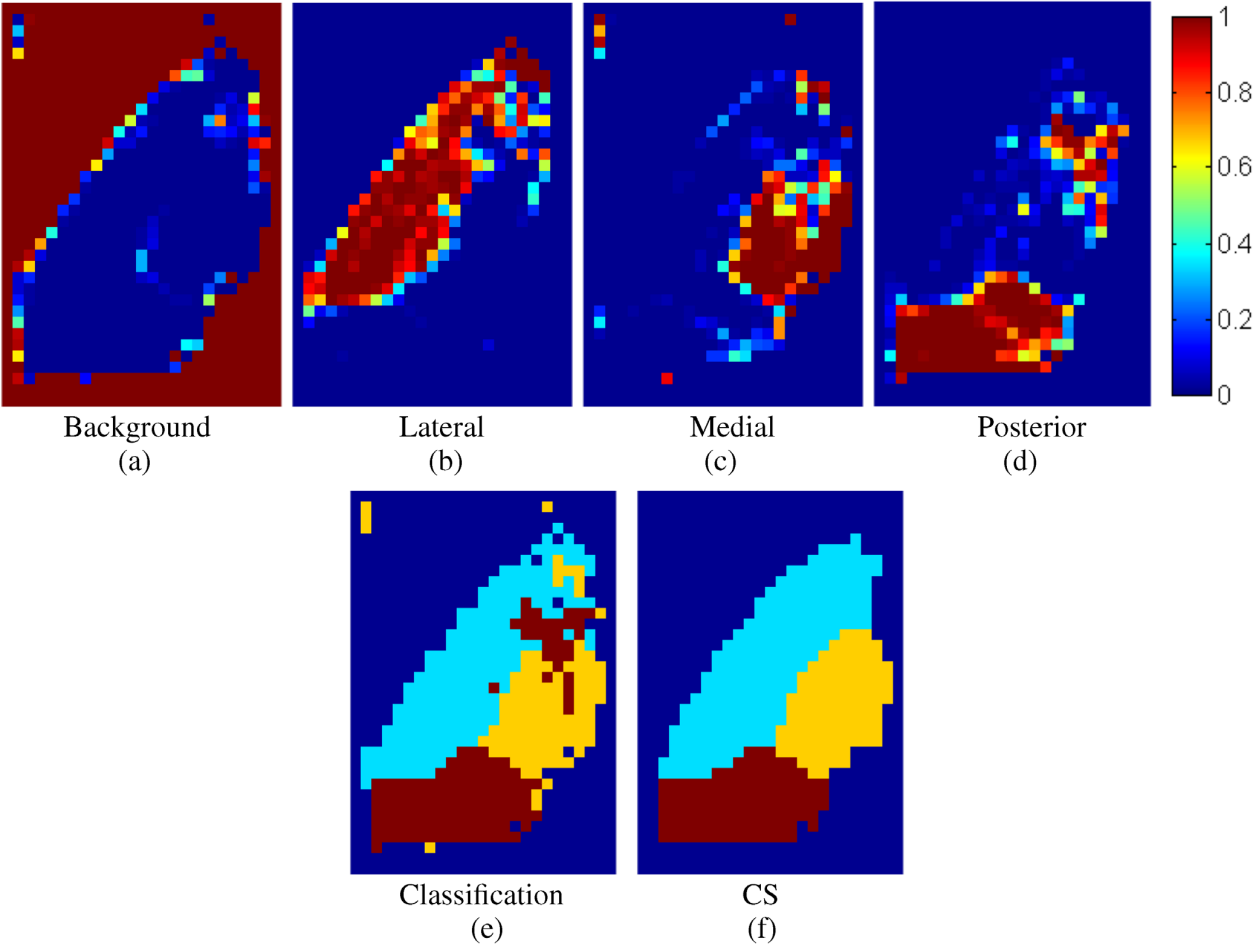
*Top row*: Posterior probabilities of the four thalamic tissue classes for the *N* = 116 template dataset obtained with Parzen classification. *Bottom row*: (left) labels derived from the posterior probabilities, and (right) refined segmentation using convex segmentation (CS) on the posterior map

In this implementation, classifiers were set out to assign four posterior probabilities per voxel, that is, those of belonging to background, lateral, medial and posterior subregions of the thalamus. Figure 4a–d shows posterior probability maps using the Parzen classifier on the average Dataset II template. The figure indicates that accurate classification of specific subnuclear groups and the background region is feasible; supporting, thus, the choice of feature space and classifier. Overall, the best performing algorithms in our preliminary assessment were *k*‐NN and Parzen classifiers (see results for other classifiers in Appendix B). For k‐NN, the optimal number of nearest neighbours, *k*, was *k* = 3. For the Parzen classifier, the empirically optimal parameter *h*, that is, the width of the Gaussian smoothing kernel, was *h* = 0.1668. Figure 4e further confirmed that the Parzen classifier output is overall in agreement with a priori knowledge on the regional distribution of subnuclear groups. However, as predicted, winner‐takes‐all local classification introduced undesirable regional discontinuities. This was substantially mitigated through the additional CS step as shown in Figure 4f.

### 3.2 General performance evaluation

#### 3.2.1 Convex segmentation validation

**Figure 5:**
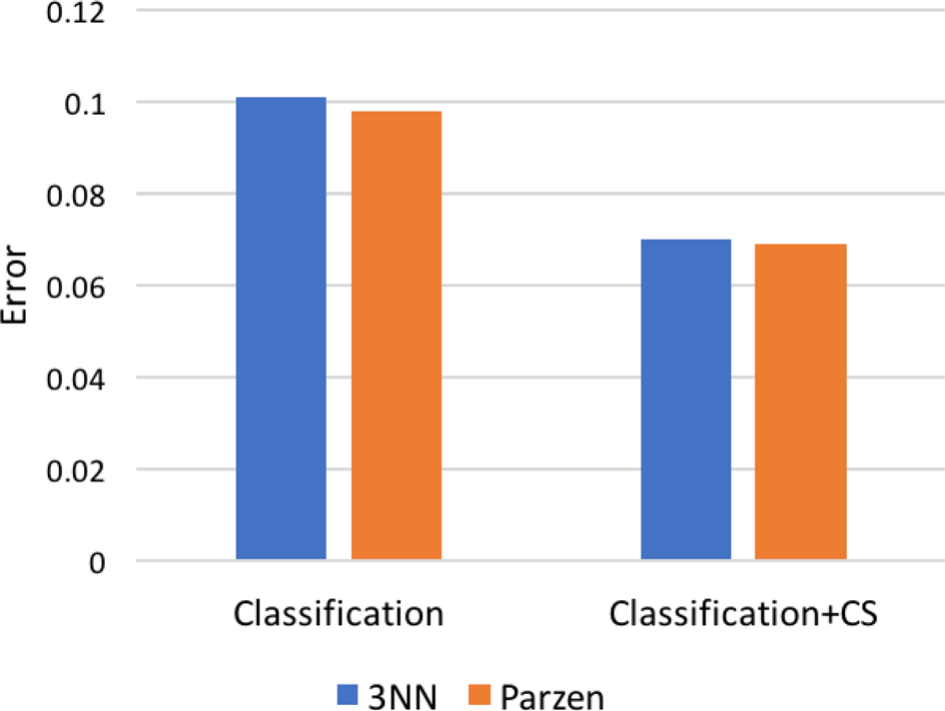
Error rates for (left) classification and (right) classification followed by convex segmentation (CS) on Dataset II average template data. Bars represent misclassification frequency, that is, overall proportion of errors relative to the manually traced ground truth

The introduction of convex segmentation systematically improved classification performance as shown in Figure 5, which, in turn, also confirmed that posterior probability maps from both *k*‐NN and Parzen classifiers are suitable pre‐conditioners for the CS formulation in (1).

#### 3.2.2 Algorithm comparison

**Figure 6:**
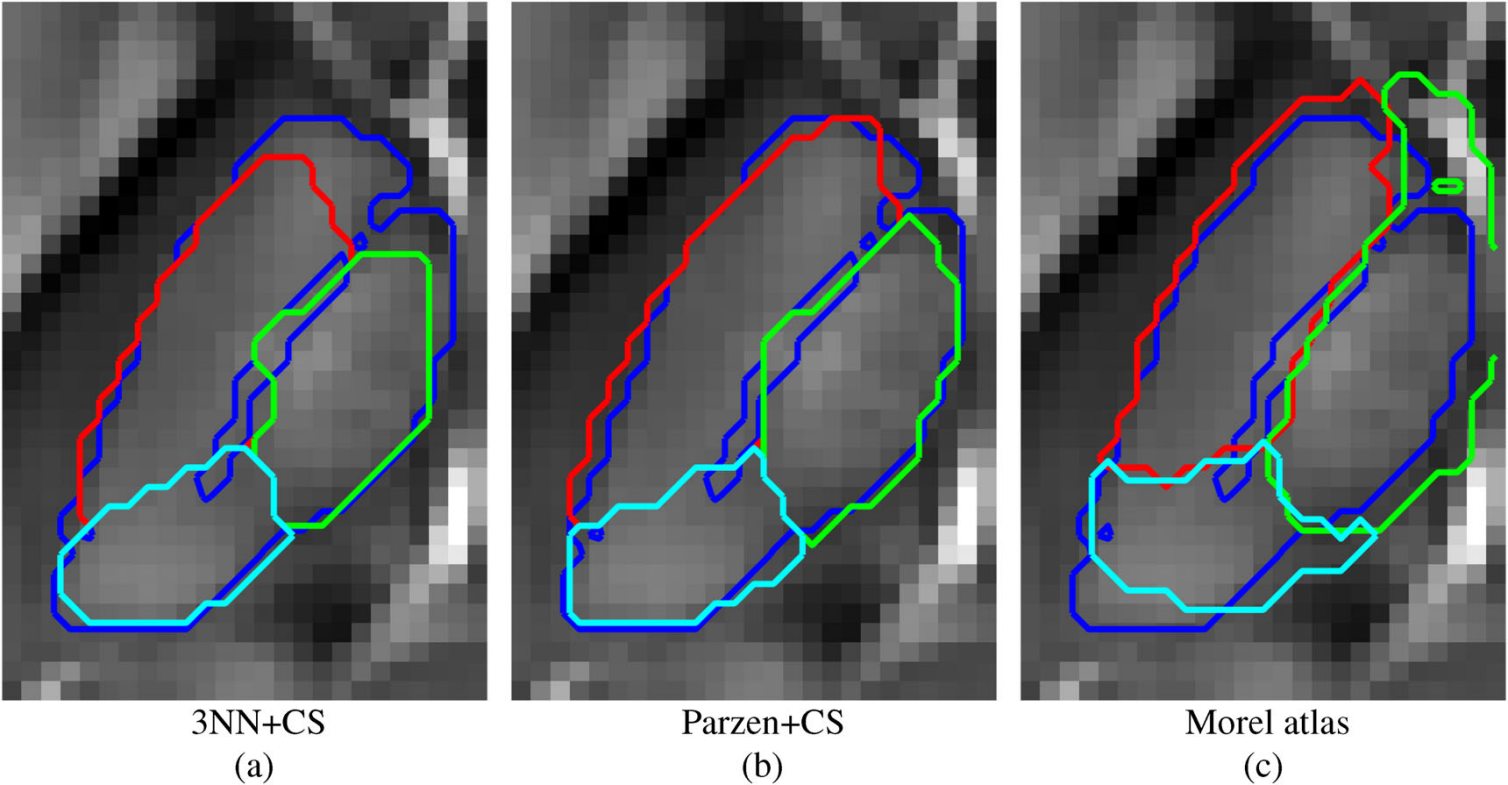
Convex segmentation results (for the template Dataset II) from different algorithmic implementations and the Morel atlas overlay onto the Dataset II QSM template. The blue overlay represents the ground truth's outer contour. Red, green and cyan contours are the results for the different approaches

Figure 6 illustrates segmentation results for all methods herein evaluated. Outputs from the proposed multi‐contrast method were in greater agreement with the manual ground truth than atlas‐based Morel segmentation, which is solely based on template co‐registration.

It is worth noting that in this particular implementation the background region outsizes (approximately 4:1) the extent of putative thalamic regions. Therefore, segmentation results are reported in Table 1 both as global classification errors and true positive rates; the latter computed for non‐background regions only. Such an evaluation confirmed the proposed algorithm outperforms Morel atlas segmentation on all performance metrics. Pre‐conditioning with 3‐NN and Parzen based posterior probabilities minimised classification error and true positive rates, respectively.

**Table 1:** Segmentation performance for the new algorithmic implementations and the standard Morel method applied on the Dataset II template

Classifiers	% global error	% TP (nuclei)
3‐NN + CS	7.0	74.8
Parzen + CS	**6.9**	**88.4**
Morel atlas	13.3	69.7

*Note*: The proposed implementations outperformed standard Morel segmentation on both performance metrics: Global error and true positive (TP) rate. The bold values indicate the best results as lowest global error and highest TP.

#### 3.2.3 Regularisation parameter selection for convex segmentation

The 3‐NN and Parzen based segmentation results in Figure 6 and Table 1 were obtained through solving the convex optimisation problem defined in (1), which has a regularisation multiplier, *λ*, that requires optimisation for optimal solution smoothness. In this study, *λ* was optimised empirically on a small subset: For 3‐NN, we chose *λ* = 1, and for Parzen *λ* = 5. We then confirmed the validity of this choice calculating overall classification errors (on the *N* = 116 template dataset) for a range of regularisation parameters. Results from this validation experiment are summarised in Table 2.

**Table 2:** Classification error as a function of classifier and *λ* parameter

3‐NN		Parzen	
*λ*	% error	*λ*	% error
0.1	9.5	4	7.3
0.5	7.0	4.5	7.2
**1**	7.0	**5**	6.9
1.5	10.0	5.5	6.9

#### 3.2.4 Algorithm performance as a function of input data type and number of contrasts provided

**Figure 7:**
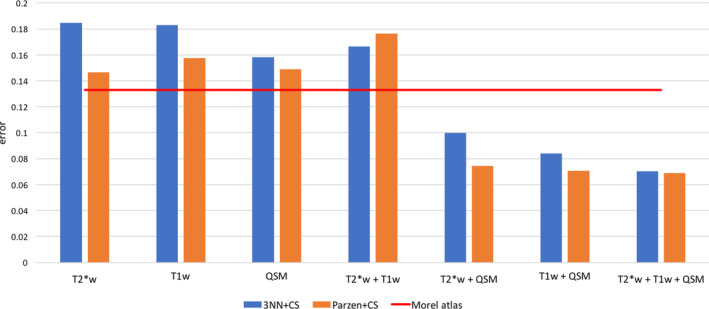
Algorithm performance comparison as a function of input MRI data for Dataset II. The red line indicates the error rate for the Morel atlas segmentation. Classification errors were greatly reduced when combining QSM with other contrasts. The global minimum error was obtained using all three contrasts

A unique aspect of the proposed algorithm is that it can integrate information from several MRI contrasts capturing simultaneously different views of the anatomy. In this study, we hypothesised that *T*_1_‐, $T_{2}^{*}$‐weighting and QSM all provide differentially relevant information about internal thalamic boundaries. In order to substantiate this claim, we estimated algorithm performance for all the available combinations, that is, one, two or three data types, using the same 27‐dimensional feature space that was previously optimised. CS errors are shown in Figure 7 for pre‐conditioning with both 3‐NN and Parzen classifiers. Interestingly, using single contrasts alone as input data led to systematically greater error rates than when using QSM in combination with other contrast types. Confirming our hypothesis, the best segmentation performance was obtained when using all MRI information. Although some differences were observed, overall both pre‐conditioning approaches, that is, 3‐NN and Parzen classification, yielded relatively similar error rates throughout.

### 3.3 Convex segmentation with additional priors for increased performance in single subjects

**Figure 8:**
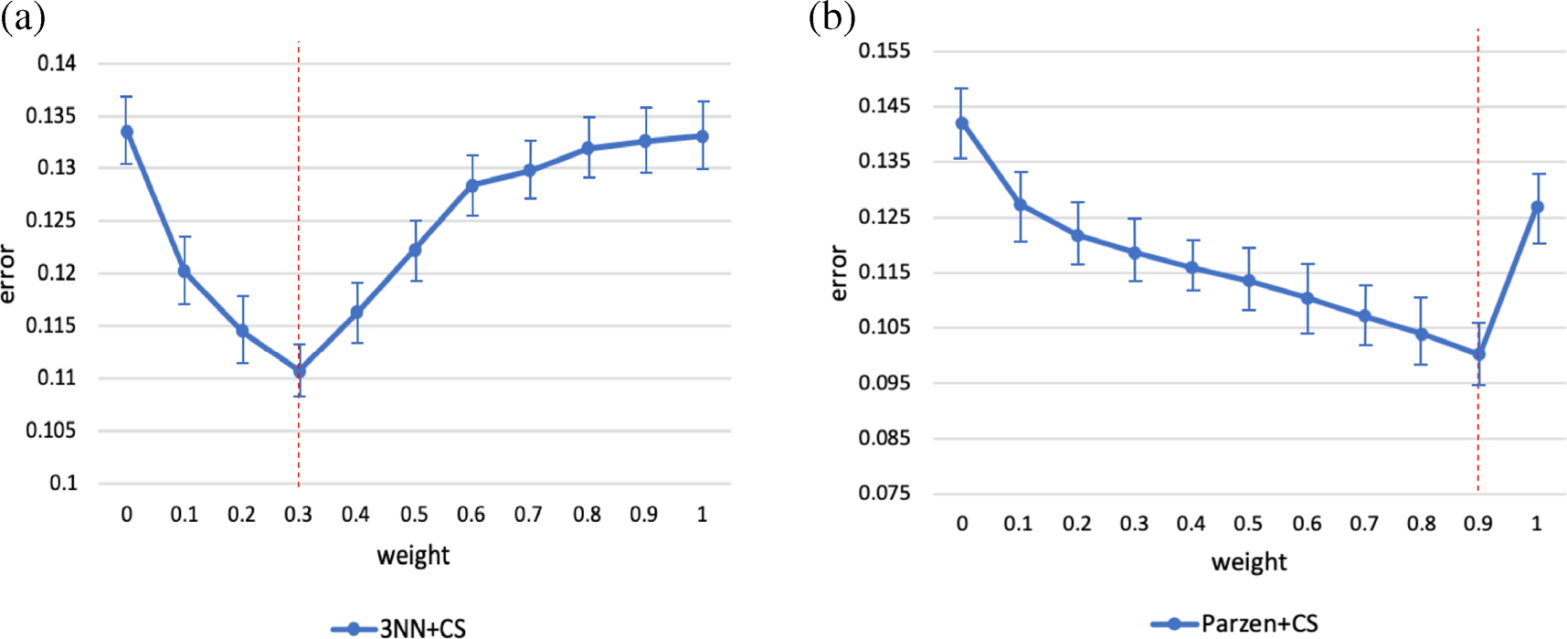
Classification error (with respect to manual gold standard) as a function of *w*. Bars denote mean error (from average Dataset II template segmentation) for each possible combination of input data. Data points and error bars denote mean and *SEM* across *N* = 12 test datasets. The red lines indicate the empirical optima *w* (*w* = 0.4 for 3‐NN and *w* = 0.9 for Parzen)

We also confirmed that constraining the data term for fidelity with training‐average tissue priors is feasible and desirable to improve accuracy and robustness in single‐subject thalamic subnuclei segmentation. The consistency weight, *w* in (2), represents a trade‐off between the calculated posterior probabilities and template‐based priors. On a *N* = 6 test subset, we explored how classification error varies as a function of *w*. This is illustrated in Figure 8, which indicated that 3‐NN is generally preferred (to Parzen based training) in this context; 3‐NN pre‐conditioning resulted in greater accuracy with optimal performance for a critical *w* = 0.4.

The primary aim of this analysis was to evaluate if performance with this extension was pre‐processing method dependent. In light of Figure 8, we can conclude that for this particular problem 3‐NN classification seems be the optimal pre‐processing method. However, further cross‐validation work is required to establish optimal *w* with greater certainty and specificity.

**Figure 9:**
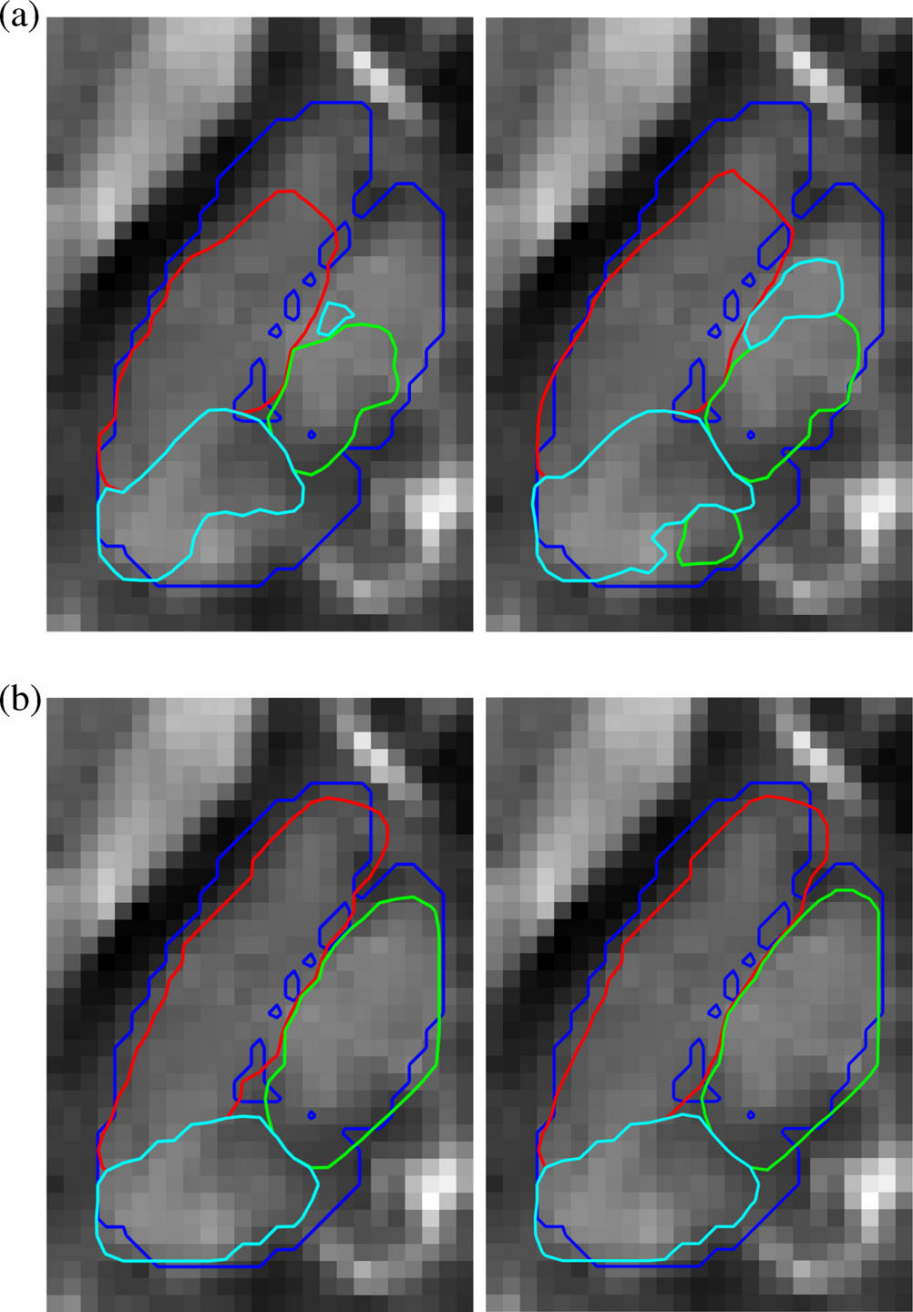
Representative convex segmentation for single‐subject data with and without training‐average priors. The blue overlay represents the ground truth's outer boundary. Red, green and cyan contours are the results for the different approaches. Contours are plotted onto the QSM contrast. (a) Convex segmentation with (left) 3‐NN and (right) Parzen based pre‐conditioners. (b) Weighted convex segmentation with 3‐NN (left, 40% prior) and Parzen based pre‐conditioners (right, 90% prior)

**Figure 10:**
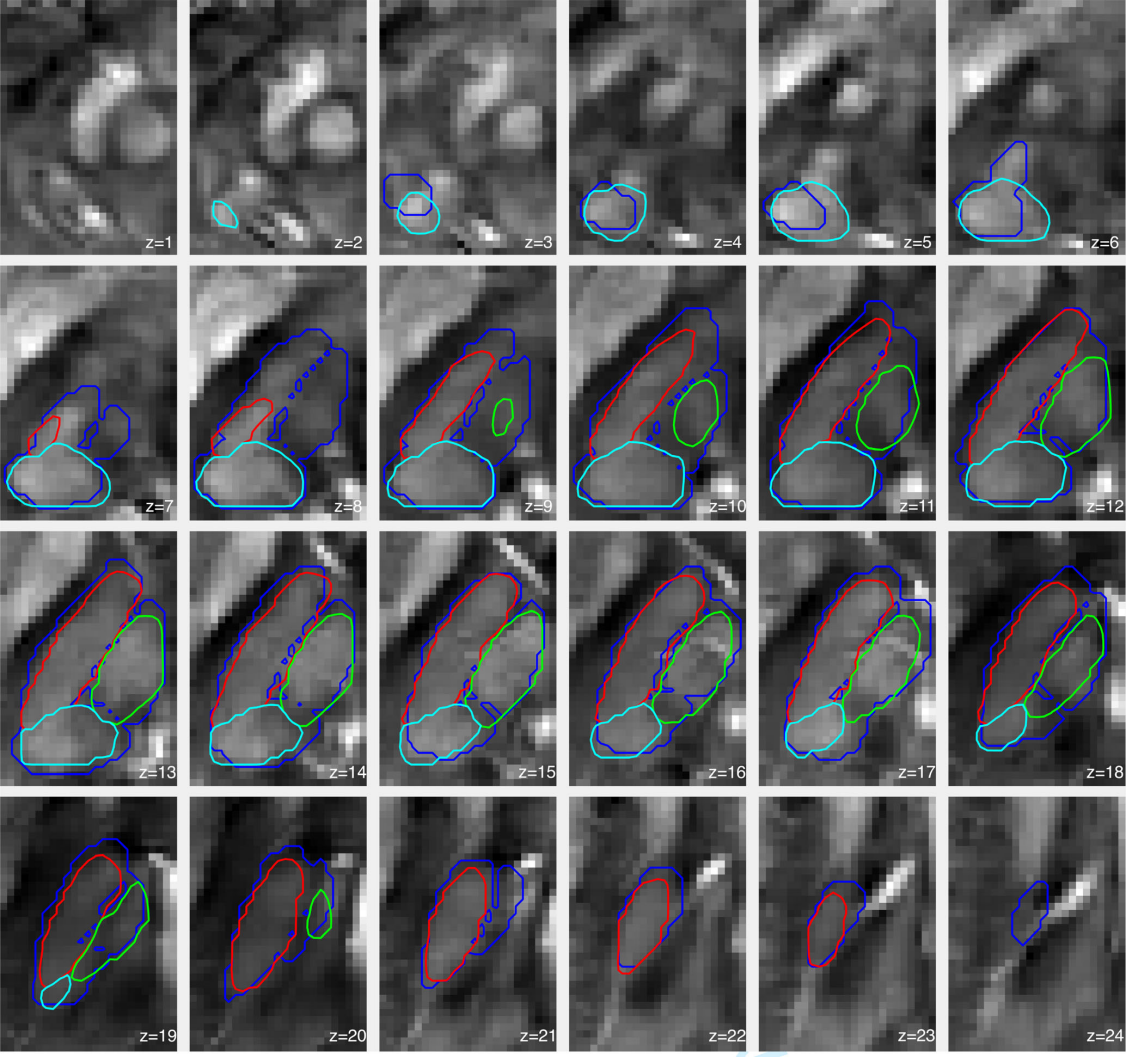
Extended view (*z*‐slices) for single‐subject thalamus segmentation using 3‐NN based pre‐processing and *w* = 0.4. The blue overlay represents the ground truth's outer boundary. Red, green and cyan contours are the segmentation results for the three subnuclear groups. Contours are plotted onto the QSM contrast

**Figure 11:**
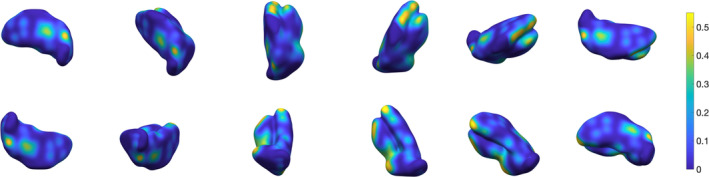
Three‐dimensional rendering of thalamus segmentation for subject #2, using 3‐NN pre‐processing and *w* = 0.4.The surface is colour‐coded by average absolute distance with respect to ground truth's outer boundary

**Figure 12:**
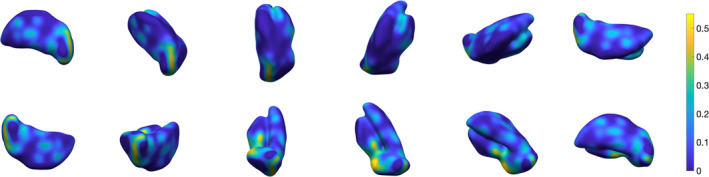
Three‐dimensional rendering of thalamus segmentation for subject #3, using 3‐NN pre‐processing and *w* = 0.4. The surface is colour‐coded by average absolute distance with respect to ground truth's outer boundary

**Figure 13:**
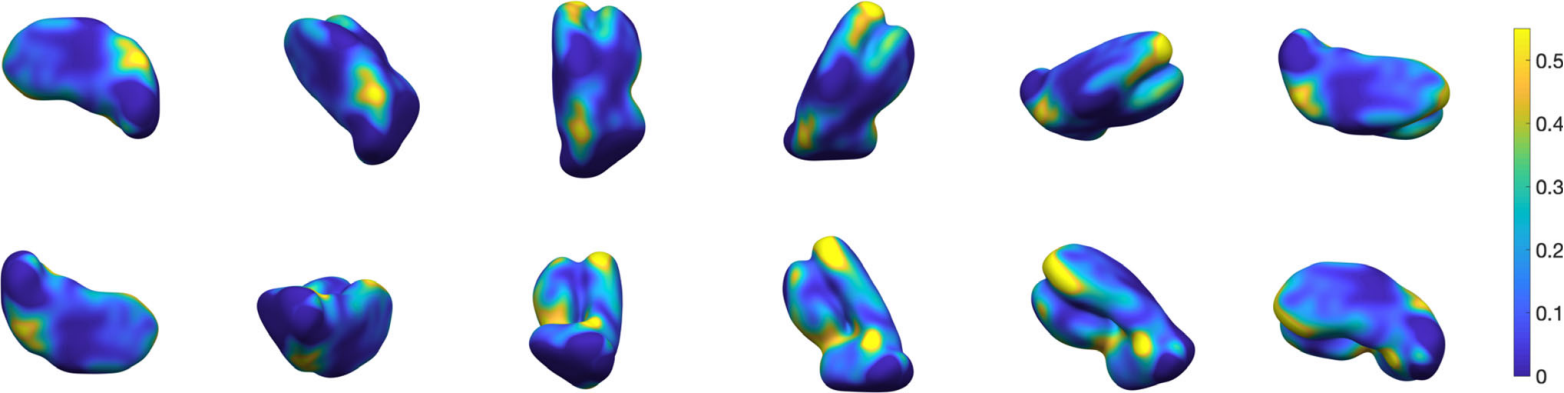
Three‐dimensional rendering of thalamus segmentation for subject #4, using 3‐NN pre‐processing and *w* = 0.4. The surface is colour‐coded by average absolute distance with respect to ground truth's outer boundary

**Figure 14:**
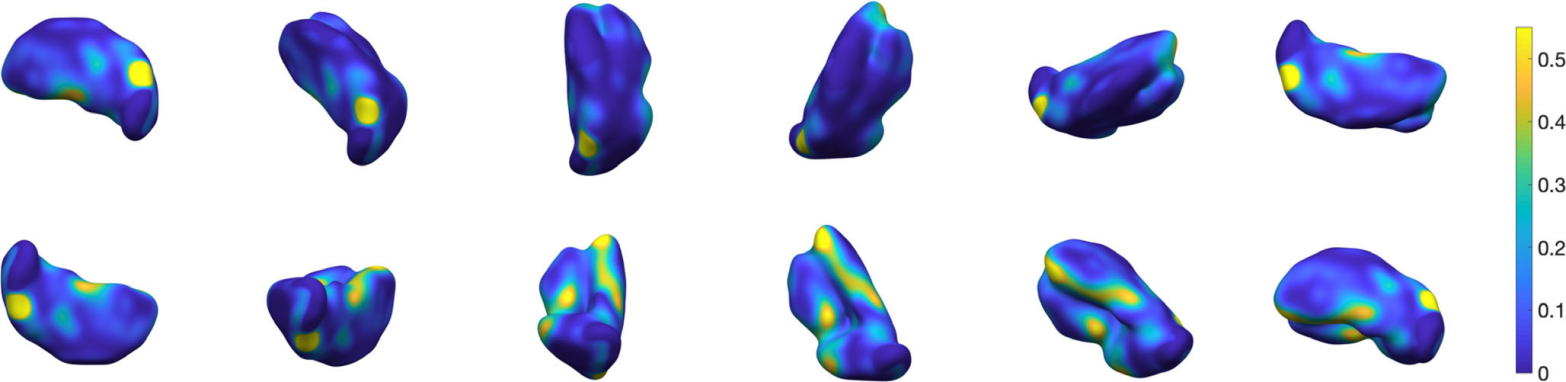
Three‐dimensional rendering of thalamus segmentation for subject #5, using 3‐NN pre‐processing and *w* = 0.4. The surface is colour‐coded by average absolute distance with respect to ground truth's outer boundary

**Figure 15:**
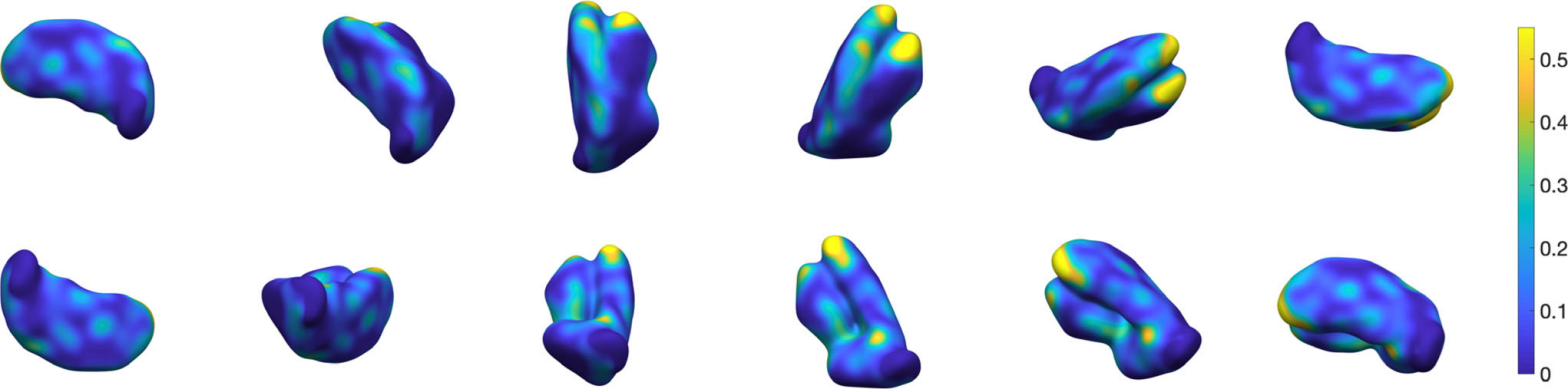
Three‐dimensional rendering of thalamus segmentation for subject #6, using 3‐NN pre‐processing and *w* = 0.4. The surface is colour‐coded by average absolute distance with respect to ground truth's outer boundary

**Figure 16:**
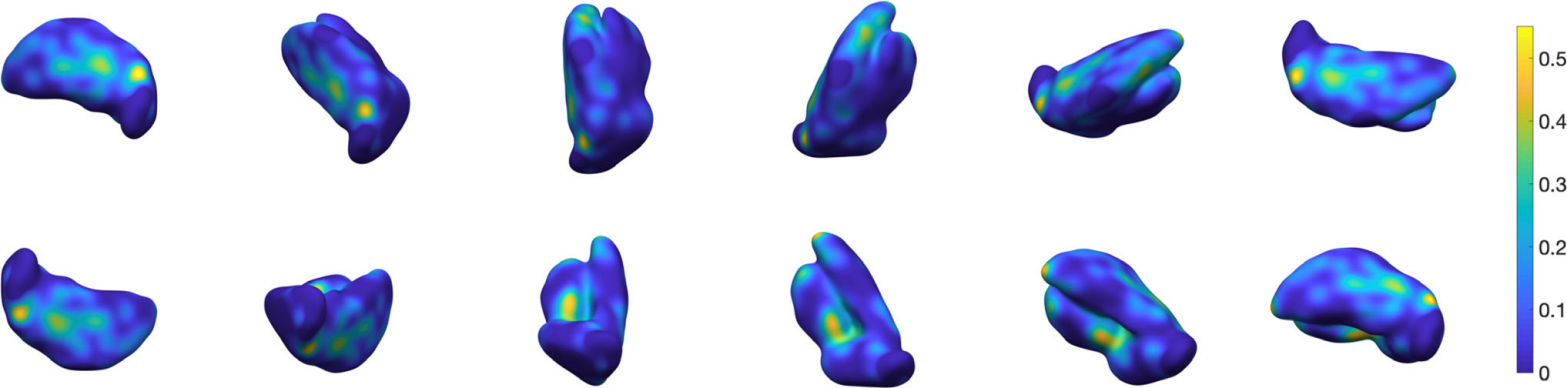
Three‐dimensional rendering of the thalamus segmentation for Dataset II (*N* = 116) average templates, using 3‐NN pre‐processing and *w* = 0.4. The surface is colour‐coded by average absolute distance with respect to ground truth's outer boundary

From a qualitative standpoint, Figure 9 illustrates the improvement in single‐subject segmentation when weighting the fidelity term by the training‐data based prior. Weighted segmentations with both supervised learning approaches (i.e., 3‐NN and Parzen) converged to solutions that were overall in close agreement with the manual ground truth. Extended results for the same subject using 3‐NN are shown in Figure 10. The remaining 3‐NN based single‐subject results are shown as 3D surface plots in Appendix D (Figures 11, 12, 13, 14, 15), which illustrate that thalamus segmentations in single subjects follow, with no exception in this small test dataset, a similar structural pattern. Segmentation results for the Dataset II template are also shown in Appendix D (Figure 16).

## 4 DISCUSSION

In this study, we present a data‐driven method to segment several internal thalamic boundaries using multi‐contrast MRI data. We had three imaging contrasts available to drive the segmentation procedure: *T*_1_, $T_{2}^{*}$‐weighting and QSM. We confirmed that using all information maximised performance, and also found evidence suggesting that QSM was the most informative contrast type for this segmentation problem. Different data types or new implementations for other anatomical regions will require new calibration work.

This work was motivated by the observation that study‐wise MRI templates obtained using highly iterative non‐linear coregistration routines are showing superb anatomical detail over and above what can be inferred from individual datasets. It is therefore unsurprising these are being used to trace regions of interest that are not available from automated segmentation tools (Acosta‐Cabronero et al., 2016; Betts, Acosta‐Cabronero, Cardenas‐Blanco, Nestor, & Dzel, 2016). Although this is an effective strategy, it assumes both that there are no co‐registration errors in the calculation of the study‐wise template space and that the manual reference is an exact definition of the region of interest, which are somewhat inaccurate assumptions. In this work, we broke away from this idealisation and propose to correct these errors with two additional steps: One of pattern recognition, followed by convex segmentation promoting (from a Bayesian standpoint) segmentation boundaries that are short, continuous and regular while also consistent with contrast variations in single subjects. Furthermore, to capitalise on the facts (a) that multiple MRI contrasts are typically acquired in the same imaging session, and (b) that different MRI contrast types could act in concert to help resolve tissue boundaries, the algorithm was implemented in multivariate form. In turn, this new method yielded regional boundaries that were in good agreement with manually traced contours. This was in stark contrast with the output from Morel atlas based segmentation of the same subnuclear groups, confirming that data‐driven approaches (such as that which is hereby proposed) signify an improvement (with respect to co‐registration based atlas labelling methods) in terms of consistency with manual segmentation. Future work is warranted to carry out additional comparisons with other state‐of‐the‐art methods (such as those cited in the introduction), and target other highly relevant deep brain nuclei.

It is also worth noting that posterior probability maps from individual datasets can be noisy. In this study, with a view to make the convex segmentation algorithm more robust in this regime, we introduced an additional data fidelity weight in (2) to enable additional prior knowledge to be transferred from the training reference to single‐subject segmentations. Such an approach led to significant improvements for both classifiers, although we noted optimal performance (i.e. lower errors with respect to the gold standard) specifically for 3‐NN based modelling and inclusion of 40% prior knowledge. Intuitively, *w*‐dependent errors reflect the complex interaction between co‐registration performance, accuracy on training‐template manual delineation and the algorithm's ability to identify biologically meaningful boundaries between tissue types. In other words, the finding that segmentation errors were systematically minimised by *w* < 1 confirmed that the proposed algorithm effectively corrects for co‐registration and/or manual initialisation errors. We cannot guarantee, however, that the proposed implementation (i.e. 3‐NN classification with *λ* = 1 and CS with *w* = 0.5) will be optimal for other regions and/or data types. This warrants further investigation. We also would like to point out that future work could also formulate the classification and convex optimisation steps as a joint problem to reduce the propagation of errors. Additionally, in future work the TV segmentation term will be extended to 3D.

An important consideration for early adopters of this method is that posterior probability maps can only be obtained from models trained on separate data. In this study, we had sufficient power to split the dataset into training and test subsets. However, future studies wanting to implement this algorithm with limited available data may consider, for example, an algorithmic extension for synthetic data augmentation.

In conclusion, this work presented a highly versatile multi‐contrast segmentation framework and its successful application to identify thalamic substructures. In practice, this method can be seen as the basis to segment any brain structure identifiable (or labelled) on any widely available template or atlas (e.g. the Ilinsky atlas (Ilinsky et al., 2018)). In addition to developing appropriate forward pipelines for bootstrapping training data augmentation, further improvements might be obtained using, for example, deep learning, which may eliminate the need for ad hoc spatial constraints.

## Data Availability

The dataset used in this work and the proposed supervised learning and convex segmentation implementations are available at \url{https://github.com/veronicacorona/multicontrastSegmentation.git}.

## APPENDIX A 1

### Classifiers in our study

In the following, we review the classifiers used in our approach, namely k‐Nearest Neighbours and Parzen classifiers (see Theodoridis and Koutroumbas (2008) for more details). We recall that in our work the dataset is built on a voxel representation, namely, each voxel in the image domain corresponds to a data point.

#### *k*‐Nearest neighbours (*k*‐NN)

The *k*‐nearest neighbours (*k*‐NN) algorithm is a non‐parametric classifier, which assigns an object to the most common class within its *k* nearest neighbours. An unlabelled data point *ψ* is located at the centre of a cell, whose volume is not fixed and will vary until *k* neighbours in the dataset of labelled examples (training set) are covered.

The *k*‐NN density estimator is

(A1) $$\hat{p}\left(\psi \right)=\frac{k}{{\mathrm{nV}}_{k}}$$

where *n* is the number of data points in the training set and *V*_k_ is the volume of the sphere centred at *ψ* with radius *r* (the distance to the k‐th nearest neighbour). Let us assume we have *n*_y_ data points of class *y* ∈ *Y*. Using the *k*‐NN density estimate (A1), we can approximate the class conditional probability

$$\hat{p}\left(\psi |y\right)=\frac{k_{y}}{n_{y}V_{k}}$$

where *k*_*y*_ are the *k* nearest neighbours to *ψ* in the training set with label *y*. The prior probability of label *y* is

$$\hat{p}\left(y\right)=\frac{n_{y}}{n}.$$

From Bayes theorem, the posterior probability is

(A2) $$\hat{p}\left(y|\psi \right)=\frac{\hat{p}\left(\psi |y\right)\hat{p}\left(y\right)}{\hat{p}\left(\psi \right)}.$$

Then, the *k*‐NN classification rule is given by the maximum a posteriori estimator which, from the above, is given by

$$\hat{y}=\arg\max_{y\in Y}\hat{p}\left(y|\psi \right)=\arg\max_{y\in Y}\frac{k_{y}}{k}=\arg\max_{y\in Y}k_{y}.$$

Therefore, given a new object *ψ*, *k*‐NN finds the closest *k* neighbours in the training set and assign to *ψ* the label of the prevailing class.

#### Parzen window classifier

In *m*‐dimensional feature space, the Parzen estimator fixes a hypercube of length *h*, and volume *h* ^*m*^ to each data point ***ψ***. We define the window function *ϕ*(***u***):

$$\phi \left(u\right)=\left\{\begin{array}{ll} 1 & |u_{j}|\leq \frac{h}{2},\;j=1,\ldots ,m\\ 0 & \text{otherwise}. \end{array}\right.$$

The window function is 1 inside the hypercube and 0 outside. Let ***ψ***_*i*_, *i* = 1, …, *n*, be the feature vector for each of the *n* data points in the training set. The number of samples in the hypercube centred at a new point ***ψ*** is

$$k=\sum_{i=1}^{n}\phi \left(\frac{\psi -\psi_{i}}{h}\right).$$

From the above, the kernel density estimator is

(A3) $$\hat{p}\left(\psi \right)=\frac{1}{h^{m}}\left(\frac{1}{n}\sum_{i=1}^{n}\phi \left(\frac{\psi -\psi_{i}}{h}\right)\right)$$

where *ϕ*(·) is the window function, also known as Parzen window. Given *n*_*y*_ data points of class *y*, from (A3) we can estimate the class conditional probability as

$$\hat{p}\left(\psi |y\right)=\frac{1}{n_{y}h^{m}}\sum_{i=1|y_{i}=y}^{n_{y}}\phi \left(\frac{\psi -\psi_{i}}{h}\right).$$

Now if we consider a Gaussian kernel for *ϕ*, from Bayes theorem (A2), the posterior probability is

$$\hat{p}\left(y|\psi \right)=\frac{\sum_{i=1|y_{i}=y}^{n_{y}}e^{-\frac{1}{2}{\left(\frac{\psi -\psi_{i}}{h}\right)}^{2}}}{\sum_{i=1}^{n}e^{-\frac{1}{2}{\left(\frac{\psi -\psi_{i}}{h}\right)}^{2}}}.$$

In the classification stage, we therefore compute a posterior probability map from which we can derive labels as

$$\hat{y}=\arg\max_{y}\hat{p}\left(y|\psi \right).$$

## APPENDIX B 1

### Experimental results in other classifiers

Before selecting k‐NN and Parzen classifiers, several other methods had been investigated in this study, including support vector machine (SVM), random forest (RF) and Naive‐Bayes (NB).

The SVM model maps samples as point in space and constructs a decision boundary as a hyperplane whose margin with the nearest training sample of any class is maximised. It can apply non‐linear classification by using the kernel trick, which represents a set of mathematical function to implicitly map inputs into high‐dimensional feature space. We show the results selecting polynomial kernels of order 3, 5 and 7.

Random Forest is an ensemble learning algorithm which merges results from many weak learners into one high quality ensemble prediction. The prediction of new objects is made by averaging predictions from all the decision trees. We chose a RUSboost algorithm and different sizes of decision trees. We show results for 100 and 500 decision trees.

Naive‐Bayes is based on the Bayes theorem and assume strong statistical independence between features. Considering independent conditional feature distributions, it reduces high‐dimensional problems to multiple one‐dimensional estimation. We used a Gaussian model to fit the data.

Implementations are from the Statistics and Machine Learning toolbox in MATLAB (Table B1).

**Table B1:** Classification errors and TP rate for rejected classifiers

Classifiers	% classification error	% TP (nuclei)
SVM order = 3	42.10	9.48
SVM order = 5	29.57	18.22
SVM order = 7	34.89	29.84
Random forest (100)	13.87	43.18
Random forest (500)	13.53	60.95
Naive‐Bayes	29.31	66.58

*Note*: SVM performances are very poor, in particular the generalisation for minor classes. RF has a relatively low misclassification error but the TP rate is not sufficient to accurately identify thalamic nuclei. NB results in bad classifications, although TP rate for nuclei class is the highest among the rejected classifiers.

## APPENDIX C 1

### Total variation

The total variation (TV) is defined as the L^1^‐norm of a discrete finite‐difference approximation of the two‐dimensional gradient (∇ : ℝ^*n*^ → (ℝ^2^)^*n*^ of *u*, that is ∇*u*(*i*, *j*) = (∇_1_ *u*(*i*, *j*), ∇_2_ *u*(*i*, *j*))^*T*^, with

$$\begin{array}{l} \nabla_{1}u\left(i,j\right)=\left\{\begin{array}{ll} u\left(i+1,j\right)-u\left(i,j\right) & \text{if}\;i<n_{1}\\ 0 & \text{if}\;i=n_{1} \end{array}\right.\\ \nabla_{2}u\left(i,j\right)=\left\{\begin{array}{ll} u\left(i,j+1\right)-u\left(i,j\right) & \text{if}\;j<n_{2}\\ 0 & \text{if}\;j=n_{2}. \end{array}\right. \end{array}$$

The total variation is then in the discrete setting

$$\mathrm{TV}\left(u\right)={\left\|\nabla u\right\|}_{2,1}=\sum_{\left(i,j\right)\in \Omega }\sqrt{{\left|\nabla_{1}u\left(i,j\right)\right|}^{2}+{\left|\nabla_{2}u\left(i,j\right)\right|}^{2}}.$$

## APPENDIX D 1

### 3D rendered surfaces of segmentation results for single subjects

Here, we show 3D visualisations of segmentation results using noisy, single‐subject test data in contrast to results from high SNR, average template data errors. It was noted that the local error mode for each subject was below 10% across the external surface. Of note, the highest segmentation errors were observed near the location of the anterior thalamic nucleus, which was an expected result due to the close proximity of large highly deoxygenated blood vessels.
